# Automatic Annotation of Unlabeled Data from Smartphone-Based Motion and Location Sensors

**DOI:** 10.3390/s18072134

**Published:** 2018-07-03

**Authors:** Nsikak Pius Owoh, Manmeet Mahinderjit Singh, Zarul Fitri Zaaba

**Affiliations:** School of Computer Sciences, Universiti Sains Malaysia, 11800 USM Penang, Malaysia; onp15_com079@student.usm.my (N.P.O.); zarulfitri@usm.my (Z.F.Z.)

**Keywords:** clustering, activity recognition, sensitive data, data security, multivariate data

## Abstract

Automatic data annotation eliminates most of the challenges we faced due to the manual methods of annotating sensor data. It significantly improves users’ experience during sensing activities since their active involvement in the labeling process is reduced. An unsupervised learning technique such as clustering can be used to automatically annotate sensor data. However, the lingering issue with clustering is the validation of generated clusters. In this paper, we adopted the *k*-means clustering algorithm for annotating unlabeled sensor data for the purpose of detecting sensitive location information of mobile crowd sensing users. Furthermore, we proposed a cluster validation index for the *k*-means algorithm, which is based on Multiple Pair-Frequency. Thereafter, we trained three classifiers (Support Vector Machine, *K*-Nearest Neighbor, and Naïve Bayes) using cluster labels generated from the *k*-means clustering algorithm. The accuracy, precision, and recall of these classifiers were evaluated during the classification of “non-sensitive” and “sensitive” data from motion and location sensors. Very high accuracy scores were recorded from Support Vector Machine and *K*-Nearest Neighbor classifiers while a fairly high accuracy score was recorded from the Naïve Bayes classifier. With the hybridized machine learning (unsupervised and supervised) technique presented in this paper, unlabeled sensor data was automatically annotated and then classified.

## 1. Introduction

Over the years, mobile crowd sensing (MCS) has revolutionized into an attractive way of gathering data [1]. Smartphones, which are examples of mobile sensing devices, are now integrated with several embedded sensors such as the accelerometer, the gyroscope, the magnetometer, a GPS, light sensors, proximity sensors, etc. These sensors gather data, which are useful in different domains [2]. MCS is applied to environmental monitoring [3], healthcare [4], and traffic monitoring. MCS plays a vital role in the actualization of smart cities, which aim at improving the quality of life of citizens using ICT tools [5]. In addition, mobile devices such as smartphones, tablets, and wearables are suitable for gathering information pertaining to the activities of users. Activity recognition in areas like healthcare [6], smart homes [7], and real-time monitoring of physical wellbeing [8] has improved significantly with the continuous advancement in sensing capabilities of smartphones. Despite the numerous opportunities and applicability of mobile sensing, methods used in labeling sensor data have remained challenging [9]. In the activity recognition domain, most data collection approaches require users to be actively involved in the labeling process [10]. This manual annotation approach requires users to be conscious when performing sensing activities since they need to provide labels to each activity (e.g., walking, running, sitting, etc.) [11]. This provides sub-optimal ‘user experience’. In addition, the privacy of users may be violated when direct observation is used as an annotation method. In place of annotation via direct observation, approaches such as experience sampling (using GUI-based apps to request for current and previous activity labels) [12], self-documentation of activities by the user (e.g., use of diaries) [13], and the hybridization of these approaches have been implemented. Albeit, using these methods to annotate a small amount of unlabeled data, these processes are still error prone. The need for ground truth labeling from experts, therefore, cannot be over emphasized [14]. However, the acquisition of accurate ground truth labeling is difficult to accomplish due to a large amount of unlabeled data from sensors. Automatic annotation offers a solution to this pressing problem of data labeling. Techniques aimed at providing automatic annotation of sensor data have been proposed mainly in the active recognition domain [14,15,16]. Unfortunately, other areas such as sensor data security and user privacy, which could also benefit from the merits of automatic data annotation, remain unexplored. Basically, users of sensing applications are always concerned with how data are collected and utilized especially if their location, speech, or private images are revealed by sensed data. Although some existing methods that are based on privacy-preserving data mining and cryptography have been proposed to tackle the problem, the issue still persists [17]. To this effect, this paper presents an approach for annotating unlabeled sensor data for the purpose of detecting sensitive location information of users during sensing activities.

In the real-world, GPS signals are not constantly available during mobile sensing especially when the user is indoors [18]. Additionally, users may deliberately turn off their GPS sensors to conserve phone battery even while using other sensors such as the accelerometer and the gyroscope. Miluzzo et al. [19] showed that, in a day, a typical smartphone user uses only 4.5% of the GPS signal. Securing sensitive location information of users is a difficult task due to the inconsistencies in the acquisition of GPS data streams. Nevertheless, classifiers (such as SVM, KNN, etc.) can be used to predict accurately when location information from the GPS sensor are received during any sensing activity. For this to happen, classifiers must first be trained using labeled data (supervised learning) [14]. This increases the need for automatic data annotation techniques.

Motivated by this, we adopt the *k*-means clustering for the automatic labeling of multivariate sensor data in order to identify location-related information of users. We believe this is the basis for effective security of sensitive data in MCS. The contributions of this paper are below.

1. Annotate unlabeled multivariate sensor data as: ‘non-sensitive’ (does not contain GPS data) or ‘sensitive’ (contains GPS data).
2. Propose a multiple pair frequency cluster validation index (MPFCVI) for evaluating generated cluster labels.
3. Evaluate the accuracy of supervised learning classifiers (SVM, KNN, and NB) using generated cluster labels.

In this paper, we use the word annotation and labeling interchangeably. The rest of this paper is structured as follows. Section 2 presents a review of related works on data annotation methods as well as cluster validation schemes. In Section 3, we present our proposed cluster validation index for the *k*-means clustering algorithm. Furthermore, datasets and methods used for clustering and training of classifiers are also discussed in this section. The results from clustering and classification of sensor data are shown in Section 4. In Section 5, we discuss obtained results and we conclude the paper in Section 6.

## 2. Related Works

With respect to machine learning and pattern recognition, we survey learning paradigms and their required data (labeled or unlabeled). We focus more on unlabeled sensor data and its appropriate annotation technique (such as clustering). Then a brief discussion on the cluster validation index is given.

### 2.1. Supervised Learning Paradigms

Presently, a number of activity recognition systems depend on the application of supervised learning on sensor data for accurate prediction of user activity [20]. Deep learning was adopted in Radu et al.; Hammerla et al.; Ronao and Cho; Ordóñez and Roggen [21,22,23,24] to accurately recognize human activities by using accelerometer sensors and gyroscope sensors on smartphones. Labeled data by users were employed for the classification task. Liu et al. [25], employed SVM in classifying data from accelerometers and GPS sensors for physical activity recognition. They showed that SVM performed better than KNN and NB in terms of accuracy.

However, employing the supervised learning approach requires labeled data [8,15]. This can be expensive in terms of annotation costs [26]. Additionally, the burden is placed on users when they are actively involved in the data labeling process. On the other hand, the privacy of users may be violated from direct observation or the use of cameras by external observers [16].

### 2.2. Semi-Supervised Learning Paradigm

Different approaches have been proposed in the semi-supervised domain in an effort to minimize the quantity of fully-labeled data needed [10]. Active learning/labeling [27,28], which is one of the most prominent approaches, detects portions of data that are best for annotation. Meanwhile, manual annotation was minimized when classifying activities in the proposed semi-supervised learning framework in Yao et al. [29]. In reducing the amount of labeled data, Ma et al.; Hong et al. [15,30] performed semi-supervised learning by dividing data into identical groups (called ‘populations’) and then fully label only a portion of each population. Guan et al. [31] proposed a graphical model based on the Auto-regressive Hidden Markov Model, which is a weakly supervised method that uses multi-instance learning. Based on mining data from mobile devices, Bhattacharya et al.; Rawassizadeh et al.; Nath; Srinivasan et al. [14,18,32,33] used semi-supervised techniques to predict human activities and behaviors from both single and multiple sensors.

Despite the small amount of labeled data required in semi-supervised learning, manual annotation is still performed either by the user or an observer.

### 2.3. Unsupervised Learning Paradigm

Unsupervised learning is often referred to as “zero resource” since prior information is not required [10]. This technique makes use of unlabeled data. Maekawa et al. [15] proposed an unsupervised learning approach for activity recognition that models activities of users by adapting to the user’s test data. Riboni et al. [16] introduced an unsupervised method for the Activity of Daily Living (ADL) and the smart home. This approach is based on ontology and probabilistic reasoning and can be applied in several environments.

Mobility is an important feature of MCS since users employ motion and location sensors to gather data while moving from one location to another. To predict a user’s location with respect to his/her activity, data from motion sensors and location sensors must be accurately annotated. In view of this, we review some proposed tools and platforms for the collection and analyzes of motion and location data in MCS.

In Cardone et al. [5], an open source platform called *ParticipAct* is proposed for sensor data collection, which is also capable of recognizing physical activities and geolocation of MCS users. Activity recognition is achieved by inferring motion related data (walking, standing still, running, biking, etc.) with location data (GPS coordinates). *ParticipAct* was tested with data collected from 300 students on the University of Bologna campus. Kiukkonen et al. [34] presented a data collection system that acquires rich location data from nearly 170 participants, which is used to study the behavior of heterogeneous populations. Outdoor trajectories of users are captured by the state machine module of the system. On the other hand, Fiandrino et al. [35] proposed *CrowdSenSim*, which is a simulating tool for both participatory and opportunistic mobile crowd sensing activities. Using this tool, the performance of MCS systems can be analyzed in realistic urban environments especially when dealing with data collection and participant recruitment. Cardone et al. [3] presented *McSense*, which is a model for generating time-variant resource maps useful in the design of crowd sensing participation for smart cities. The model was validated on a large dataset by testing its prediction accuracy on sensing tasks. The reliability and flexibility of the framework is based on its distributed architecture and data analysis functionalities. Lastly, APISENSE (https://www.inria.fr/en/centre/paris/innovation/rii-telecoms-du-futur/demos/apisense-r-crowd-sensing-made-easy) is a middleware platform, which employs sensors in smartphones for the collection and sharing of large but relevant data while maintaining the privacy of users. These large datasets are stored in the cloud using web standards and can be accessed by the appropriate stakeholders.

Unfortunately, these studies do not provide the data annotation method used during data collection. This further justifies the need for a study that provides a detailed annotation process for data from motion sensors and location sensors.

Some researchers have made efforts in developing tools, models, and approaches that automatically annotate sensor data. Others have worked on minimizing efforts and the cost of manual annotation. Most of these works focus specifically but not exclusively on activity recognition. However, applying data annotation (be it manual or automatic) in the areas of sensor data security and user privacy remain unexplored. In this paper, we show how an unsupervised learning technique (e.g., clustering) can be used to annotate unlabeled sensor data for the identification of sensitive data.

Clustering algorithms (*k*-means, EM, etc.) have the ability to group together data with high similarities, which generates cluster labels [36]. This technique has produced reasonable results in areas such as trajectory annotation [37,38] and speech recording [39] among others. However, validating results from clustering algorithms still remains a major challenge [40,41]. Cluster validity techniques are formal measures (quantitative and objective) used in evaluating cluster analysis [42]. 

A number of cluster validation techniques have been proposed by other researchers. In Yu et al. [43], an extended DTRS (Decision Theoretic Rough Set) validity scheme for evaluating the quality of clustering analysis was proposed. The validation index is based on the between-cluster separation and the within-cluster scatter principle, which considers compactness and separation between clusters. To reduce the computational cost of cluster validity and to eliminate the reliance on the dimension of the feature vector, Cui et al. [44] proposed a novel validity index, which is based on Pairing Frequency. This cluster validation technique was detailed for fuzzy *c*-means (FCM) clustering. The validation index is based on logical reasoning and statistical analysis of pairwise patterns and assembles several partition outputs together using the global information from data. However, few attempts have been made on validating clusters generated from the *k*-means algorithm.

## 3. Materials and Methods

In this section, we present Multiple Pair-Frequency Cluster Validation Index (MFPCVI) for the *k*-means algorithm. Furthermore, the dataset as well as the experimental procedures used for both clustering and classification of sensor data are also discussed in this section.

### 3.1. k-Means Clustering

The *k*-means algorithm is an unsupervised learning algorithm commonly used in tackling clustering problems in sensor networks due to its simple implementation and linear complexity [36]. It separates data into different groups (referred to as clusters). With *k*-means clustering, cluster centers (C) are stochastically initialized to k from points in a given data to ensure uniqueness of all centroids (i.e., $\forall \;centroids\;C_{i}\;and\;C_{j},\;C_{i}\neq C_{j}$). For the *k*-means to function, three parameters must be provided by the user, which are: number of clusters k, cluster initialization, and the distance metric [42]. However, the number of cluster k is the most important of the three parameters. *k*-means can be formally represented by the equation below. 

Let $D=\left\{d_{1},\ldots ,d_{n}\right\}$ be the data (sensor data), $\mu_{q}=\sum_{d\epsilon C_{q}}(d|n_{q})$ be the centroid of the cluster $C_{q}$, and let K be the cluster number (1≤q≤K). Then the objective function of the *k*-means clustering algorithm is the sum of the squared error (SSE) shown below.

(1) $$S_{k}=\overset{K}{\underset{q=1}{\sum }}\underset{d\epsilon C_{q}}{\sum }\;\;\;\;∥d-\mu_{q}{∥}^{2}$$

where $\mu_{q}$ is the mean of cluster $C_{q}$ containing data points $\left\{d_{1},\ldots ,d_{n}\right\}$ and d is a high dimension set of observations. The aim here is to minimize the objective function for a fixed number of clusters (k=2).

### 3.2. Multiple Pair-Frequency Cluster Validation Index (MPFCVI)

In this subsection, we present the proposed Multiple Pair-Frequency Cluster Validation Index (MPFCVI). The aim of the proposed model is to validate clusters generated from the *k*-means algorithm when employed to group sensor data based on high similarity and logical reasoning. Mathematical notations used in the formulation of the validation index is shown in Table 1.

The proposed validation index for *k*-means is an extension of the Pairing Frequency Cluster Validation Index proposed in Cui, Zhang [44] for Fuzzy *c*-means (FCM). 

To start with, a matrix of members in which $A=\left[A_{ij}\right]$ is obtained and this can be gotten from the result of a *k*-means clustering, which is shown in Equation (1) above.

(2) $$\;A_{c\times r}=\left[\begin{array}{ccc} A_{11} & \ldots & A_{1r}\\ \vdots & \vdots & \vdots \\ A_{c1} & \ldots & A_{cr} \end{array}\right]$$

Each object can be defined as $D_{j}=\left[A_{ij}\right]$ to obtain the value of i when $A_{ij}$ (1≤i≤c) is the highest value. From this, we derive multiple pairs of the object as $S_{a}$, $S_{b}$ (1≤a,b≤r) to be members of the same cluster based on the value of c *iff* $S_{a}$ = $S_{b}$ while the other pair of objects $S_{f}$, $S_{g}$ (0≤f,g≤r) to be in the same cluster (different from that of $S_{a}$, $S_{b}$) and also based on the value of c if $S_{f}$ = $S_{g}$ and $((S_{f}$, $S_{g})\neq (S_{a}$|$S_{b}))$.

Just like with FPCVI [44], we define the value of c to be defined by the equation below.

(3) $$T_{c}=\left[\begin{array}{ccc} t_{11} & \ldots & t_{1r}\\ \vdots & \vdots & \vdots \\ t_{r1} & \ldots & t_{rr} \end{array}\right]$$

The element of $T_{c}$ is represented by $t_{ag|pm}$ which explains to a large extent the possibility of $S_{a}$ = $S_{g}$ belonging to the same cluster and $S_{p}$ = $S_{m}$ in another cluster. Using the features (F), which is a set of attributes of the sensors and is represented by (X, Y, Z, L, V), we define MPFCVI by the equation below. The algorithm in Table 2 validates generated clusters (“non-sensitive” and “sensitive”).

(4) $$t_{ab|fg}=\{\begin{array}{c} 0;\;if\;\left(\left|X\right|\cdot \left|Y\right|\cdot \left|Z\right|\right)*\left(\left|L\right|.\left|V\right|\right)<1\;\forall \;S_{a}=S_{b}\\ 1;\;if\;\left(\left|X\right|\cdot \left|Y\right|\cdot \left|Z\right|\right)+\left(\left|L\right|.\left|V\right|\right)\geq 1\;\forall \;S_{f}=S_{g} \end{array}$$

### 3.3. Dataset

The smartphone-based multivariate dataset [45] used for our experiment consists of raw sensing data together with a timestamp of each sample. The requirement of the dataset was that it should be collected using multiple sensors (such as an accelerometer, a gyroscope, a magnetometer, and GPS) that gather data concurrently. In addition, the data collection process was to simulate a real-world scenario of using motion sensors and location sensors both in outdoor and indoor environments. The ‘travel’ dataset was collected while driving (outdoor) and having stopovers (indoor) within a duration of 518 min. The dataset consists of 8000 instances and 11 attributes. After feature selection, we had the following: accelerometer (Ax, Ay, Az), gyroscope (Gx, Gy, Gz), magnetometer (Mx, My, Mz), and GPs (latitude, longitude). Similar to datasets in Cardone et al.; Kiukkonen et al. [3,5,34], the dataset used for our experiment consists of data from motion sensors and location sensors. A Samsung Galaxy S4 with an embedded accelerometer, a gyroscope, a magnetometer, and GPS sensors was used to gather motion and location related data. The availability/unavailability of GPS data with respect to outdoor and indoor movements of users are reflected in the dataset, which is why it was chosen over other publicly available datasets. Since the aim of this work was to automatically annotate and predict sensitive GPS data when motion and location sensors are used in MCS, dataset [45] was deemed most suitable.

A brief explanation of the sensors, which were used to acquire data recorded in the dataset is given below.

A. Sensor Data from Accelerometer

**Figure 1:**
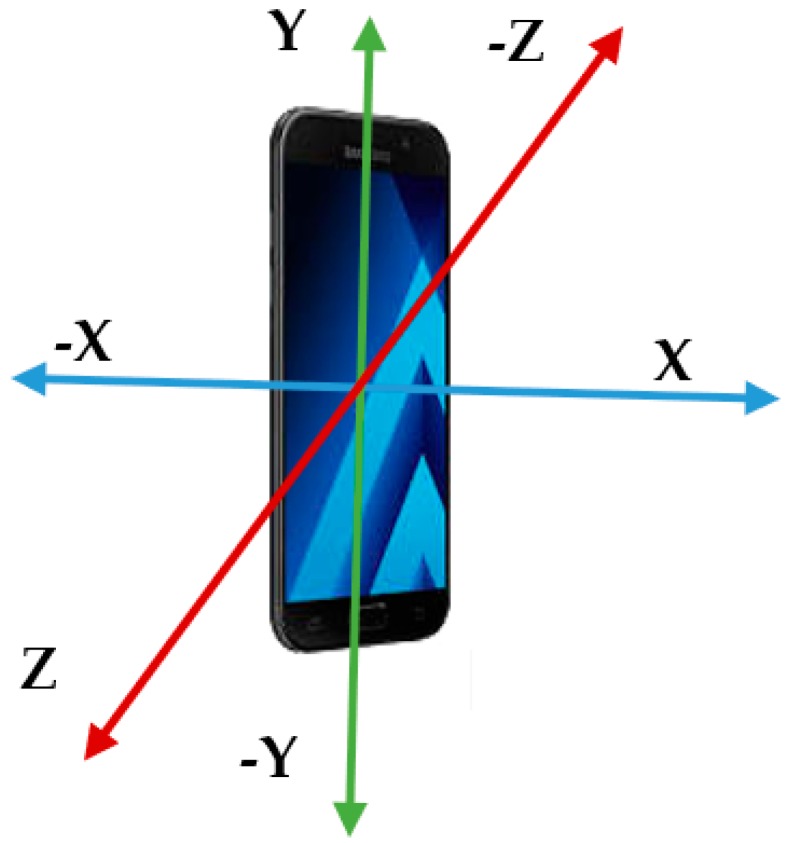
Three-dimensional axes of an accelerometer.

Accelerometer sensor data uses the *X*, *Y*, *Z* axis to sense motion and acceleration, which is shown in Figure 1. It detects gravity when movement is sensed in various directions. When a forward movement is made, the accelerometer captures backward pressure and then accurately detects a forward movement. Similarly, when a backward movement is made, the accelerometer gets the forward pressure to detect a backward movement. The same principle holds for both upward and downward movements. Acceleration captured by smartphones are measured by [$m/s^{2}$].

B. Gyroscope

**Figure 2:**
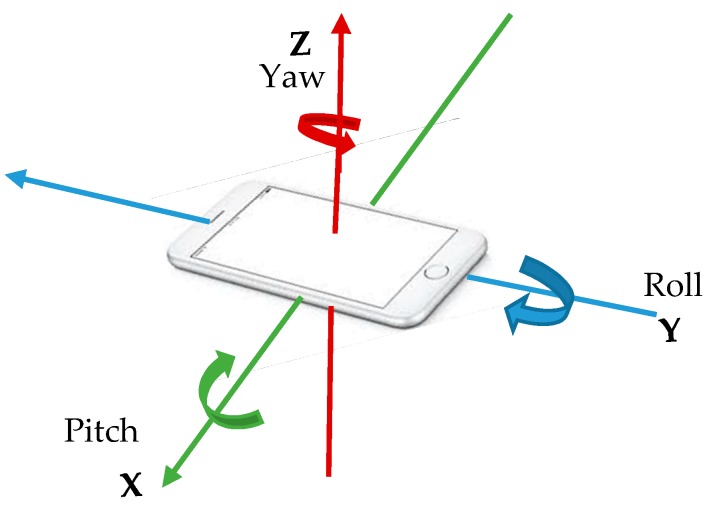
Three-dimensional axes of a gyroscope.

Gyroscopes identify orientation information such as pitch, roll, and yaw rates using the *X*, *Y*, *Z* axis as shown in Figure 2. Unlike accelerometers, they do not detect force but add precision to data from the accelerometer. Gravity g=0 when the pitch or yaw is unchanged (i.e., no tilt or turn is made). It measures velocity by using [rad/s]. One major setback of the gyroscope sensor is bias, which is depicted using nonzero output data (with respect to a stationary device).

C. Magnetometer

The magnetometer sensor in a smartphone measures magnetic fields and detect location (north, south pole, etc.) by changing its voltage output to the smartphone. Smartphones offer raw magnetometer data together with a computed compass bearing. This measures the strength of the earth’s magnetic field, which is expressed in tesla [T]. Its application is evident in rotating maps, interfaces, and graphics based on its bearing. Data from this sensor is represented by using the (*X*, *Y*, *Z*) axis.

D. Global Positioning System (GPS)

The GPS sensor in a smartphone records the latitude and longitude to pinpoint any location on earth. This sensor detects the location of a user through the fixed distance from a satellite. Then the distance from the second satellite is measured to obtain an overlap. Basically, the received data contain streams of spatio-temporal (*x*, *y*, *t*) points. For these raw data to be used by applications, data streams are converted into finite subsequences referred to as trajectories [46].

### 3.4. Experimental Procedures

The objective of the experiment was to generate two distinct clusters by grouping together data from similar sensors (cluster-then-label). Using this automatic data annotation technique, we aimed at identifying sensitive data (from GPS sensors), which are labelled as ‘cluster 1’. Thereafter, the proposed cluster validation index, which is based on multiple pair frequency, was employed to mathematically prove the accuracy of the generated clusters. Then generated cluster labels from the *k*-means algorithm were used to train three classifiers (SVM, KNN, and NB). The entire experiment (both clustering and classification phases) were implemented in python programming language using the SciKit learn library. A breakdown of the processes involved in the experiment are discussed below.

Clustering (Cluster-then-label)

After preprocessing, clustering was performed using the *k*-means algorithm, which was run iteratively to obtain the best cluster result (clusters 0 and 1). The clusters were generated based on the concept of dissimilarity between pairs of observations. The dissimilarity in our experiment relates to the availability/unavailability of location information in each observation. The first cluster consists of observations without location information (from the GPS sensor) and were labelled ‘0‘ while the second cluster consists of observations with location information of the user and were labelled ‘1’.

Classification (Supervised Learning)

In this phase, generated cluster labels (0 and 1) were used to train and then test three classifiers (SVM, KNN, and NB). These clusters represent the ‘non-sensitive’ and ‘sensitive’ class, respectively. 

Training Phase

The major task in the training phase was to identify optimal parameters for the classifiers and to fit generated models. We used a percentage split of 25% (random state = 1, in python) as the test option, which allocated 6000 instances (75%) as training data and 2000 instances (25%) as test data. We ensured that all features provided information for the classification process. Class label used in this phase were the *k*-means generated clusters (0 and 1). The aim was to train classification models, which will accurately predict (using the test data) sensor data into either ‘class 0’ or ‘class 1’.

Validation and Test Phase

For validation, 25% of the entire dataset were used to evaluate the performance of the three classifiers (SVM, KNN, and NB). The main aim was to identify the classifier with the highest classification accuracy. The class labeled as ‘1’ was considered “sensitive” because it contains location information recorded from the GPS sensor. Correct classification of observations containing GPS data into ‘class 1’ were recorded as true positive (TP). Incorrect classification of observations without GPS data into ‘class 1’ were recorded as a false positive (FP). Furthermore, the correct classification of observations without GPS data into ‘class 0’ were counted as a true negative (TN) while the incorrect classification of observations containing GPS data into ‘class 0’ were recorded as a false negative (FN). This process was iteratively performed for all classifiers. Obtained results are shown in Section 4.

## 4. Results

**Figure 3:**
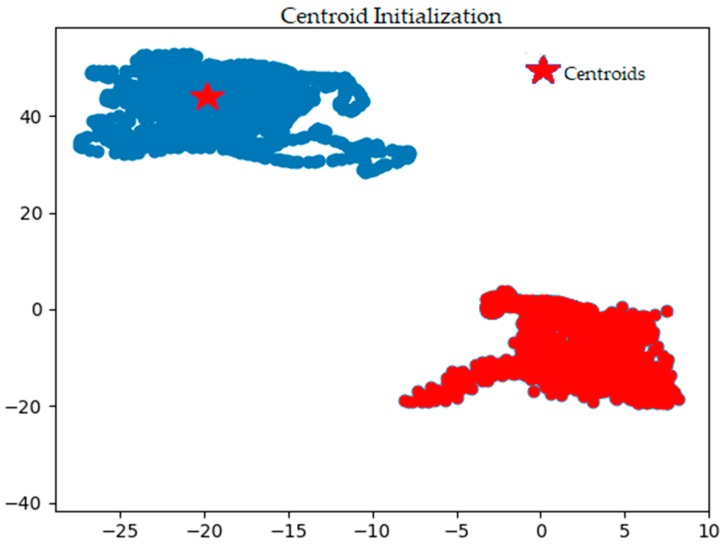
Centroid initialization of non-sensitive and sensitive clusters.

**Figure 4:**
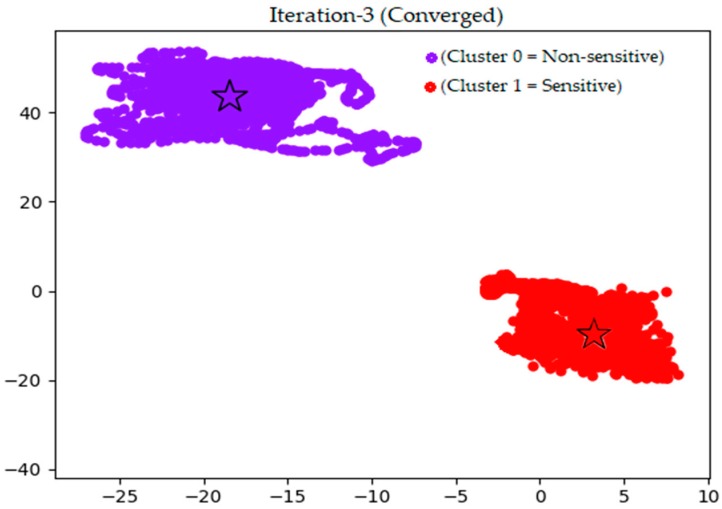
Converged non-sensitive and sensitive clusters.

Figure 3 shows the *k*-means centroid initialization using the random method. This result was obtained by calculating the distance of each feature set (data from the sensors) to the centroids using the Euclidean distance. New centroids were generated from the mean of all feature sets in each cluster. This process was iterative until no new centroids were obtained (i.e., centroids are no longer moving). A visualization of this process is captured in the change of position of (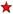) in Figure 3 to its new position in Figure 4. The different color shades and shapes of clusters seen in Figure 3 and Figure 4 show the iteration process where new members are added to each cluster before the actual convergence, which is shown in Figure 4.

Figure 4 illustrates the two clusters generated by the *k*-means algorithm after converging on the third iteration. As desired, obtained clusters satisfy the compactness and isolation property of accurate clustering [40]. ‘Cluster 0’ is a group of observations without location information from the GPS sensor and is referred to as the “non-sensitive” class. On the other hand, ‘cluster 1’ depicts grouped observations with location information from the GPS sensor (latitude and longitude) and we name it the “sensitive” class. The compactness of each cluster justifies their similarity while their dissimilarity is shown by their isolation. With this clear, ‘cluster 1’ can easily be detected as the cluster of interest with respect to sensitive data, which could reveal the user’s location. Next, we present results from the evaluation of the three classifiers (SVM, KNN, NB) and discuss their performances based on the summarized confusion matrix shown in Table 3. Elements in the confusion matrix are matched to the obtained results from each of the tested classifier. Based on the notion that the class labeled ‘0’ is the non-sensitive class, which does not contain location information of the user. The class labelled ‘1’ is the sensitive class, which contains location information of the user. We present the following.

True Positive (TP) = Correctly annotated observations containing GPS data as ‘1’ (sensitive class)
True Negative (TN) = Correctly annotated observations without GPS data as ‘0’ (non-sensitive class)
False Positive (FP) = Incorrectly annotated observations without GPS data as ‘1’ (i.e., wrongly classified into sensitive class)
False Negative (FN) = Incorrectly annotated observations containing GPS data as ‘0’ (i.e., wrongly classified into non-sensitive class)

From the class distribution of the test data, which is (class 0: 1014) and (class 1: 986), we highlight the performance of each classifier. SVM correctly classified 976 data samples containing GPS data into ‘class 1’ (TP). It also classified correctly 1008 data points that contained no location information from GPS sensor into ‘class 0’ (TN). A total of 10 data samples that do not contain GPS data were incorrectly classified into ‘class 1’ (FP). Only 6 data samples that actually contain GPS data were incorrectly classified into ‘class 0’ (FN) by SVM. 

KNN on the other hand, correctly classified 973 data samples containing GPS data into ‘class 1’ (TP). Additionally, it classified correctly 1005 data points that contained no GPS data into ‘class 0’ (TN). A total of 13 data samples that do not contain GPS data were incorrectly classified into ‘class 1’ (FP). Additionally, nine data samples that actually contain GPS data were incorrectly classified into ‘class 0’ (FN) by KNN. NB correctly classified 965 data samples containing GPS data into ‘class 1’ (TP). In addition, it classified correctly 909 data points that contained no GPS data into ‘class 0’ (TN). A total of 21 data samples that do not contain GPS data were incorrectly classified into ‘class 1’ (FP). Additionally, 105 data samples that actually contain GPS data were incorrectly classified into ‘class 0’ (FN) by NB. The A summary of the evaluation results from all three classifiers is presented in Table 4.

SVM recorded the highest accuracy of 99.3% with the lowest false negatives of six data samples. KNN and NB had an accuracy score of 98% and 94%, respectively. The aim is to identify a model that minimizes the number of observations that actually contain GPS data but are classified as “non-sensitive” (‘class 0’). Without doubt, the high accuracy scores from these classifiers is attributed to the fact that employing clustering for automatic data annotation generates accurate and reliable clusters. Unlike with manual annotation where the labeling accuracy depends solely on the user, automatic data labeling is systematic, consistent, and reproducible. 

In the information security domain, location information of users are classified as sensitive since users’ can be profiled using such information. To ensure the privacy of users when sensing activities, their location information must remain confidential and can only be accessed by authorized persons. This can be achieved using security mechanisms such as encryption and authentication. However, for these schemes to be effective and trivial, the data of interest (i.e., location information from GPS sensor) must be accurately annotated and classified. Based on the high accuracy scores obtained from evaluated classifiers in this paper, implementing this framework in smartphones will enable accurate detection of location information of users each time GPS data is received. 

**Figure 5:**
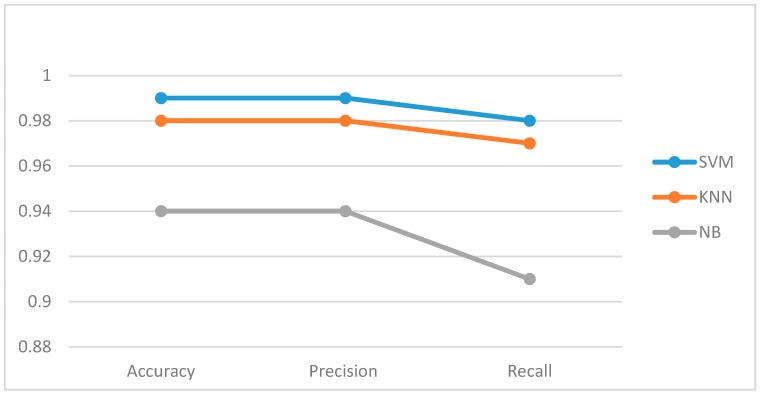
Comparison of accuracy, precision, and recall results from evaluated classifiers.

**Figure 6:**
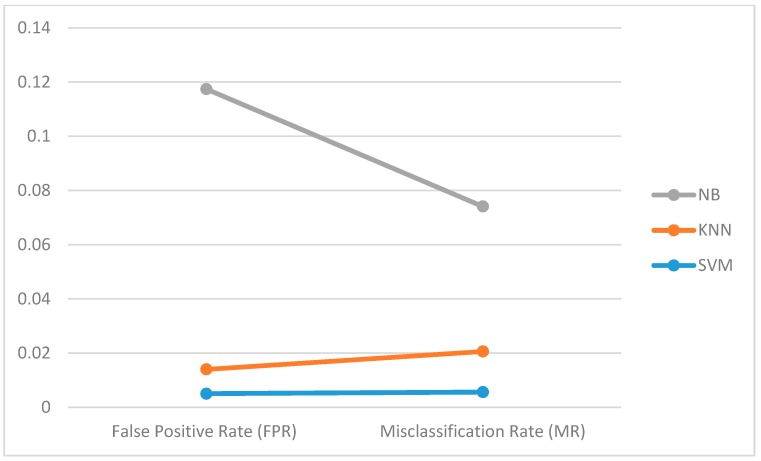
Comparison of FPR and MR results from evaluated classifiers.

Figure 5 compares SVM, KNN, and NB with respect to accuracy, precision, and recall. SVM and KNN both recorded high precision and recall score while the recall score of NB was remarkably low. Figure 6 presents the False Positive Rate (FPR) and the Misclassification Rate (MR) of the three classifiers. The noticeable difference between the three lines in Figure 6 shows the high misclassification rate recorded by Naïve Bayes classifier. The least misclassification was recorded from an SVM classifier, which justifies its high accuracy score.

**Figure 7:**
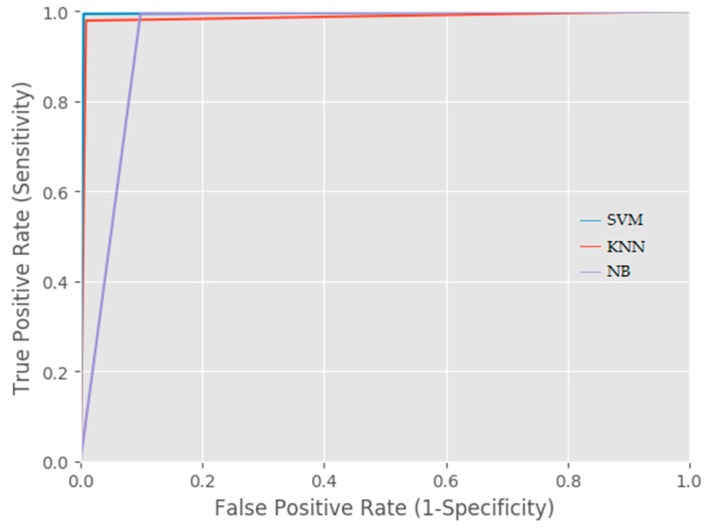
ROC Curve of SVM, KNN, and NB classifiers.

Figure 7 shows the ROC (Receiver Operator Characteristics) curve of all three classifiers (SVM, KNN, and NB) using the default threshold of 0.5. The generated ROC curves from the three classifiers present SVM and KNN as perfect classifiers. This is in line with the fact that SVM labeled the highest number of observations (i.e., 976 instances) that actually contain GPS data as ‘1’ (positive class). KNN was second best since it labelled 973 observations that contain GPS data as ‘1’. The NB classifier was the least likely since it labelled 965 observations containing GPS data as ‘1.’ Therefore, the shift in its curve moves away from 1.

## 5. Discussion

Automatic data annotation eliminates issues associated with manual annotation of data. With this method of labeling, there is no burden on the user when performing sensing activities. Smartphones simply capture sensor data in an opportunistic manner with no active user involvement in the data collection process. An unsupervised learning technique (such as clustering) can be used for automatic labeling of sensor data without prior knowledge of the dataset. We have employed the *k*-means algorithm in annotating unlabeled multivariate data from smartphone-based motion and location sensors. 

However, one major issue with clustering as pointed out in Jain [42] is the problem of cluster validation. In an effort to tackle this problem, we proposed a cluster validation index for *k*-means algorithm, which is based on multiple pair-frequency. The proposed model validates the accuracy of generated clusters employed for data annotation as well as guarantees data reliability. The Cluster Validation Index is effective in ensuring that the similarity between cluster members are high while also establishing the dissimilarity between non-cluster members. We used the generated cluster labels to train supervised classifiers (SVM, KNN, and NB). From results in Section 4, SVM had the highest accuracy of 99.3%. KNN and NB had accuracies of 98% and 94%, respectively. 

Without generality, related works presented in this paper focus on approaches and techniques in annotating sensor data for activity recognition. In this paper, we used automatic annotation for a different purpose. We proved that classifiers can be trained to predict sensitive location information of users each time GPS data is received during sensing. We further showed that obtaining labeled data to train class classifiers can be done automatically using clustering. Effective security of sensitive data is achieved when the data to be secured are accurately identified. Typically, implementing security mechanisms such as encryption of large data is non-trivial. For instance, encrypting data obtained from a navigation app like ‘waze’ can be very difficult due to the heterogeneity of sensor data. However, with classifiers, identifying sensitive data each time they are received is feasible. Automatic labeling comes in handy in this scenario since manually obtaining the ground truth would be hard to achieve. Accurate classification of sensitive data enables the implementation of encryption schemes only on the data of interest (DOI), which minimizes the computational resources used in achieving effective security. Consequently, the privacy of MCS users is preserved when their location information is effectively secured. 

Similar to works in Cardone et al.; Kiukkonen et al. [3,5,34], our proposed framework analyzes data from motion and location (accelerometer, gyroscope, magnetometer, and GPS) sensors. However, these works focus on building tools, models for either identifying user’s behaviour, and location or activity. Apart from these works, we rather detect sensitive data related to GPS coordinates of users through automatic annotation of unlabeled data from motion and location sensors. Furthermore, we compared classifiers based on their prediction accuracies before adopting the most efficient classifier, which was not done in the aforementioned works.

## 6. Conclusions

In this paper, we implemented automatic annotation on a multivariate sensor data using the *k*-means clustering algorithm. Clustering of sensor data was performed in order to group and then label observations either as “non-sensitive” (contains no location information from GPS sensor) or “sensitive” (contains location information from GPS sensor). Additionally, we proposed a Multiple Pair-Frequency Cluster Validation Index (MPFCVI) for the validation of clusters generated from the *k*-means algorithm. Emphasis of the proposed validation index was on proving mathematically the dissimilarity between pairs of observations (i.e., observations with no GPS data and observations with GPS data). The generated cluster labels (0 and 1) were used to train three classifiers (SVM, KNN, and NB). Classification results from the three classifiers showed that SVM performed slightly better than KNN with an accuracy score of 99.3% and 98%, respectively. Other evaluation metrics such as precision and recall also showed a slight edge of SVM over KNN. Comparatively, the NB classifier was the least performing classifier with an accuracy score of 94%. The results obtained is in line with earlier results presented by Liu, Gao [25], which showed that SVM performs better than KNN and NB when trained with cluster labels generated from the *k*-means algorithm. Though they only employed data from accelerometer and GPS sensors, we have presented in this paper that SVM still performs better than KNN and NB even when more sensors are used.

The experiment performed in this paper proves that the unsupervised learning technique can be used for accurate annotation of unlabeled sensor data. This approach can be useful in many ways. For instance, when implementing security mechanisms on sensor data, accurate data annotation provides insights on the “data of interest.” Furthermore, we showed that automatic data annotation can also be used in the information security domain where sensitive data such as location information of a user can be labeled without user’s active involvement. This approach improves the ‘user experience’ during sensing activities. Furthermore, this method of automatically annotating sensor data enhances the accuracy of classifiers during prediction.

## Figures and Tables

**Table 1:** Mathematical notations.

Symbol	Definition
D	Data
n	Total number of samples
K	Total number of clusters
S	Observations
F	Features (X, Y, Z, L, V)
|*X*|	Absolute value of X
|*Y*|	Absolute value of Y
|*Z*|	Absolute value of Z
|*L*|	Absolute value of L
|*V*|	Absolute value of V
·	Dot Operator

**Table 2:** Algorithm for the proposed *k*-means validation index.

**Algorithm to validate *k*-means clusters**
**Input:** The dataset N *is* the total number of instances; $K_{1},K_{2}$ the number of clusters.
1. Choose the number of clusters $\left(K_{1},\ldots ,K_{n}\right)$ 2. Randomize the initialization of clusters (K) 3. Assign each data point to the closest centroid 4. Update centroid position to the average of all assigned data points to the centroid 5. Repeat 3 6. Initialize X, Y, Z, L, V for validation
7. For (|X|·|Y|·|Z|)∗(|L|.|V|)<1, set $K_{1}$ as 0
8. *Else if*
9. (|X|·|Y|·|Z|)+(|L|.|V|)≥1, set $K_{2}$ as 1
10. *End if*
**Output:** The validated clusters $K_{1},K_{2}$ of unlabeled datasets from multiple smartphone sensors

**Table 3:** Confusion Matrix from SVM, KNN, and NB classifiers.

SVM Classifier			KNN Classifier			NB Classifier		
N = 2000	Predicted Class 0 (Non-sensitive)	Predicted Class 1 (Sensitive)	N = 2000	Predicted Class 0 (Non-sensitive)	Predicted Class 1 (Sensitive)	N = 2000	Predicted Class 0 (Non-sensitive)	Predicted Class 1 (Sensitive)
Actual Class 0 (Non-sensitive)	TN = 1008	FN = 6	Actual Class 0 (Non-sensitive)	TN = 1005	FN = 9	Actual Class 0 (Non-sensitive)	TN = 909	FN = 105
Actual Class 1 (Sensitive)	FP = 10	TP = 976	Actual Class 1 (Sensitive)	FP = 13	TP = 973	Actual Class 1 (Sensitive)	FP = 21	TP = 965

**Table 4:** Summary of Results from SVM, KNN, and NB classifiers.

Classifiers	Accuracy	Prediction Mean		Precision	Recall	False Positive Rate (FPR)	Misclassification Rate
		0 s	1 s				
**SVM**	99.3%	0.5125	0.4875	0.9948	0.9848	0.0050	0.0056
**KNN**	98%	0.5070	0.4930	0.9790	0.9700	0.0090	0.0150
**NB**	94%	0.5070	0.4930	0.9430	0.9019	0.1034	0.0535
